# Signaling Pathways and Downstream Effectors of Host Innate Immunity in Plants

**DOI:** 10.3390/ijms22169022

**Published:** 2021-08-21

**Authors:** Jitendra Kumar, Ayyagari Ramlal, Kamal Kumar, Anita Rani, Vachaspati Mishra

**Affiliations:** 1 Bangalore Bioinnovation Centre, Life Sciences Park, Electronics City Phase 1, Bengaluru 560100, India; director@bioinnovationcentre.com; 2 Division of Genetics, Indian Agricultural Research Institute (IARI), Pusa Campus, New Delhi 110012, India; ramlal.ayyagari@gmail.com; 3 School of Life Sciences, Jawaharlal Nehru University, New Delhi 110066, India; kk120293@gmail.com; 4 Department of Botany, Dyal Singh College, University of Delhi, Delhi 110003, India; anitarani@dsc.du.ac.in

**Keywords:** plant immunity, defensive pathways, signaling mechanisms, PTI, ETI, phytohormones

## Abstract

Phytopathogens, such as biotrophs, hemibiotrophs and necrotrophs, pose serious stress on the development of their host plants, compromising their yields. Plants are in constant interaction with such phytopathogens and hence are vulnerable to their attack. In order to counter these attacks, plants need to develop immunity against them. Consequently, plants have developed strategies of recognizing and countering pathogenesis through pattern-triggered immunity (PTI) and effector-triggered immunity (ETI). Pathogen perception and surveillance is mediated through receptor proteins that trigger signal transduction, initiated in the cytoplasm or at the plasma membrane (PM) surfaces. Plant hosts possess microbe-associated molecular patterns (P/MAMPs), which trigger a complex set of mechanisms through the pattern recognition receptors (PRRs) and resistance (R) genes. These interactions lead to the stimulation of cytoplasmic kinases by many phosphorylating proteins that may also be transcription factors. Furthermore, phytohormones, such as salicylic acid, jasmonic acid and ethylene, are also effective in triggering defense responses. Closure of stomata, limiting the transfer of nutrients through apoplast and symplastic movements, production of antimicrobial compounds, programmed cell death (PCD) are some of the primary defense-related mechanisms. The current article highlights the molecular processes involved in plant innate immunity (PII) and discusses the most recent and plausible scientific interventions that could be useful in augmenting PII.

## 1. Introduction

The interaction between the plants and the microbes antedate history, and they face each other constantly for various purposes, such as in the form of biocontrol agents [1], arbuscular mycorrhiza [2,3] and as many other mutual beneficiaries or pathogens since their origin. Several of these microorganisms cause various diseases in different crop plants creating havoc and enormous economic loss by compromising crop productivity and yield [4,5]. This field of plant microbes and their interactions with the plants has been an interesting emerging area of research currently. Concomitantly, this information provide useful insights on the emergence of diseases, the occurrence of genetic changes, and underlying defensive mechanisms in both plants and microbes and their effective management practices [4,5]. The current review provides an overview of the existing state of knowledge in the area of plant innate immunity (PII) and updates on the recent information that have been added to the various aspects of PII currently discussed. Furthermore, the importance of signaling pathways and their downstream effectors associated with PII in the light of recent research is discussed that is supposed to unfold vistas for new research designs for effective management of plant diseases suited for commercial utilization.

An attack by microbial pathogens, pests and tissue and cellular damage in plants, generally is detected by cell-surface receptors through the evolutionarily conserved innate immune system. According to Jones and Dangi [6], these microbial pathogens capable of impairing plant growth and reproduction respond to infection using a two-branched innate immune system that firstly recognizes and responds to molecules common to many classes of microbes, including non-pathogens and secondly, to pathogen virulence factors, either directly or through their effects on host targets. The plant immune systems (PII) and the associated pathogen molecules provide enormous insights into molecular recognition, cell biology and evolution across the biological kingdom. Their details are currently highlighted in the following sections.

## 2. Pattern-Triggered Immunity (PTI)

Pattern-triggered immunity (PTI) is the first layer in the immune response. In this process, the pattern recognition receptors recognize the conserved molecular patterns like lipopolysaccharides, peptidoglycans, chitin, flagellin, EF-Tu, DNA and ergosterol known as pathogen-associated molecular patterns (PAMPs) or microbe-associated molecular patterns (MAMPs), which aids in the hydrolysis and activation of signaling pathways including production of reactive oxygen species (ROS), MAP kinase activation and transcriptional induction of pathogen-responsive genes. Several excellent reviews of MAMPs are available [7,8,9,10]. The microbial and pathogen-associated molecular patterns are slow processes as they evolve over a considerable period [6]. One key aspect of the definition of PAMPs and MAMPs is that they are conserved and widely distributed within a class of microbes [11]. Sometimes, as a result of pathogen attack, the plants recognize that their peptides are continuously synthesized. These are released into the extracellular space, including plant apoplast, from their normal location due to damage (trauma), and these molecules are referred to as damage-associated molecular patterns (DAMPs) [12,13,14]. The MAMPs are derived from microorganisms, while DAMPs are host cell-derived and both initiate and perpetuate innate immune responses [14]. Of the DAMPs, the largest and best-characterized are polypeptides/peptides produced from larger precursor proteins that include three families discovered by Ryan and his colleagues during their study to identify systemin—a term “used to describe polypeptide defense signals that are produced by the plant in response to physical damage and induce defense genes, either locally or systemically [15]. An 18 amino acid (aa) polypeptide was isolated from a tomato seedling that was shown to induce the synthesis of wound-inducible proteinase inhibitor proteins [16]. Located in vascular parenchyma cells, the tomato systemin is generated by wound-induced processing of a 200 aa prohormone prosystemin and induces the neighboring companion cells and sieve elements of the vascular bundle to synthesize jasmonic acid (JA), that activates the expression of proteinase inhibitor genes [17]. A third family of peptide-based DAMPs, discovered in *Arabidopsis*, are 23 aa plant elicitor peptides (Peps) that are derived from a 92 aa precursor [18]. The receptors identified are known as AtPeps, which induce a variety of innate immune responses and enhanced resistance, and a form of precursor ProPep3 PROPEP3 was recently shown to be released into the extracellular space upon infection of *Arabidopsis* with hemibiotrophic *Pseudomonas syringae* [19]. A maize ortholog, *Zm*Pep1, was subsequently identified and shown to enhance resistance to microbial pathogens [20].

Extracellular ATP (eATP) comprises yet another class of plant DAMPs found in both plants and animals. Deciphering plasma membrane-localized receptors, eATPs were ascribed to signaling functions. Based on the observations on the *dorn1* [20] mutant and wound-inducible genes, eATPs have been designated as a plant DAMP [21]. Additionally, eATP is found to induce typical innate immune responses that include cytosolic Ca^2+^ influx, MAPK activation, and induction of some dense-associated genes that are involved in the biosynthesis of JA and ethylene [21]. However, it is unclear yet whether it contributes to resistance to pathogens.

A constitutive basal immunity present in plants is triggered by the pathogens, thus providing complete or incomplete resistance to them against those phytopathogens [14]. The plants have two protective physical barriers called the cuticle and cell wall, and additionally, the production of various antimicrobial compounds act as a control measure. Even though the cuticle protects against phytopathogens and pests, some fungi are able to penetrate, while the cell wall aids in protecting against various abiotic and biotic stresses [22]. There are many ways through which the phytopathogens get into the plant system and cause damage to them, such as through natural openings like stomata, lenticels, hydathodes and nectarthodes, or through wounds and cuts that occurred as a result of herbivory, rains/storms or human interventions [22]. Therefore, the plants recruit many cell-surface and intracellular immune receptors to perceive a variety of immunogenic signals associated with pathogen infection and followed by the activation of defensive signaling cascades [23].

### 2.1. Bacteria

Flagellin is the most studied protein subunit, constituting the bacterial flagellum and its receptor is a leucine-rich repeat that behaves like kinases (LRR-RLKs) FLAGELLIN SENSING 2 in *Arabidopsis*. The N terminal of the flagellin contains a 22-amino acid conserved region, which initiates and elicits flagellin sensing responses thereon [24]. Recently, it has been reported that glycosidase β-galactosidase 1 (BGAL1) acts on the glycosylated flagellin having a terminal modified viosamine, as normally flagellin is glycosylated, acting upstream of proteases in the plant apoplast, which is the site for invading bacteria’s released immunogenic peptides [25]. Therefore, bacterial strains like *Pseudomonas syringae* produce unrecognizable glycans called BGAL1-insensitive, which surpass the detection by the FLS2 [24,25,26,27].

Similarly, another LRR-RLK, bacterial elongation factor Tu (EF-Tu) receptor (EFR) detects the 18-amino acid region of the EF-Tu also initiates the signaling cascade. Upon recognition, they instantaneously heterodimerize with the LRR-RLK family coreceptor BAK1 [5,28]. Rapidly phosphorylation of the receptor-like cytoplasmic kinase (RLCK) BIK1 and its homolog PBL1, which is associated constitutively with FLS2/EFR, and BAK1 occurs and is thereby released from the receptor complexes upon MAMP perception [28,29]. This BIK1 then directly phosphorylates the plasma membrane NADPH oxidase RBOHD (respiratory burst oxidase homologue protein D), resulting in the production of reactive oxygen species (ROS) and aids in stomatal immunity which along with calcium signaling-mediated RBOHD regulation. This production of ROS is crucial for the establishment of a successful immune response against pathogens. The RBOHD is regulated by ubiquitination and C- terminal phosphorylation. It was recently reported that when AVRPPHB Susceptible1 (PBS1)-like kinase 13 (PBL13) receptor-like cytoplasmic kinase phosphorylates at the C terminal of the RBOHD at positions S862 and T912, it provides stability and affects its activity [5,28,30,31].

The effectors of bacterial pathogens generally target the kinases of the plants namely RLK and RLCK. For instance, type III effectors of *Pseudomonas syringae* including AvrPto, AvrPtoB, HopF2, and HopB1 target BAK1 while effectors of *Xanthomonas oryzae* Xoo2875 targets the BAK1 homolog of *Oryza sativa* (OsBAK1). The formation of the FLS2-BAK1 complex is interrupted by the AvrPto and AvrPtoB as they bind to BAK1. Similarly, *P. syringae* AvrPphB and *X. campestris* AvrAC target BIK1. This AvrPphB is a cysteine protease that degrades PBS1-like kinases, like BIK1 while the uridylyl transferase AvrAC phosphorylates for the activation loop of BIK1. This shows that inhibiting the kinases of the plants is beneficial and advantageous to bacterial pathogens [32].

### 2.2. Fungi

Irieda and co-workers (2019) [32] reported a novel core effector PAMP, which is highly conserved in the filamentous fungi, named necrosis-inducing secreted protein 1 (NIS1). This effector targets the RLK (BAK1) and RLCK (BIK1) kinases and thereby induces PTI signaling in the plants. This effector was first reported [33] from the *Nicotiana benthamiana*, as it caused cell death due to the pathogen, cucumber anthracnose fungus, *Colletotrichum orbiculare*. This is involved in the suppression of multiple PTI responses in the *Nicotiana benthamiana* by suppressing oxidative burst and hypersensitive responses followed by the pathogen signatures. Similarly, the fungus, *Magnaporthe oryzea* NIS1 (acquired through horizontal transfer) also suppresses some responses of PTI whereas the homolog of NIS1 in *Colletotrichum tofieldiae* (a root endophyte) shows similar responses by suppressing oxidative burst in *Nicotiana benthamiana*. The NIS1 is conserved in the Ascomycota and Basidiomycota and suggests that it is inherited and sustained through generations. Recently, it was reported [34] that the moss, *Physcomitrella patens* is able to detect chitin and thereby activates RLK-CERK1 (chitin elicitor receptor kinase 1; chitin receptor in moss) responses. Thus, the RLK-dependent PAMP recognition is inherited ancestrally in plants [32].

The rice chitin elicitor-binding protein (CEBiP) contains extracellular LysM motifs for binding chitin, but an intracellular kinase domain is absent. With the help of RNAi, it was shown that CEBiP is required for chitin-induced defenses in rice. The CERK1 of *Arabidopsis* contains three LysM motifs in the extracellular domain and an intracellular Ser/Thr kinase domain is required for perception of chitin and bind directly to chitin in vitro. It is anticipated that CERK1 forms a heterodimer with CEBiP to bind chitin. Surprisingly, it was found that CERK1 plays an important role in disease resistance to *P. syringae* bacteria raising the possibility that it also mediates the perception of an unknown bacterial PAMP [35,36,37,38,39].

### 2.3. Virus

Virus-derived nucleic acids (VDNA) may also activate PAMP recognition receptors and VDNA-PAMPs have been reported to elicit the Nuclear Shuttle Protein-Interacting Kinase 1 (NIK1)-mediated antiviral signaling pathway that transduces an antiviral signal to suppress global host translation [40]. The classical plant PTI similarly restricts virus infection as compared to non-viral pathogens, such as first undergoing the preactivation of PTI with non-viral PAMPs conferring resistance to virus infection, indicating that PTI-induced immune responses confer protection against viruses [41], and second, suppressing PTI by the pathogens in order to successfully colonize a host [40].

## 3. Effector-Triggered Immunity (ETI)

During the process of evolution, plants have developed R (resistance) proteins that can identify some of the many effectors produced by the pathogens in order to activate the defense mechanisms. Table 1 lists some of the major effectors produced by pathogens and their R genes. The foundation stone was laid by the concept given by Flor (1971) [42] for the determination of receptor–effector recognition [43]. The Flor concept for gene-for-gene hypothesis states that for each resistance gene in the host, there is a corresponding gene for avirulence in the pathogen conferring resistance and vice versa [42]. The effectors which are recognized by the R proteins are termed avirulence (Avr) proteins, and the pathogen that contains this is called an avirulent pathogen. These R proteins are primarily intracellular nucleotide binding-leucine rich repeat (NB-LRR) proteins and can be categorized based on the presence of variable N terminal coiled-coil and toll/interleukin 1 receptor-like protein families (CC or TIR NB-LRR) [44]. The coiled-coil NB-LRR (CNL) are found generally in both monocot and dicot whereas, the latter on TIR NB-LRR (TNL) is found only in the dicots [39]. Both pattern recognition receptors and NLRs, initiates the downstream signaling networks thereby leading to the expression of defense-related genes, production of reactive oxygen species (ROS) and callose deposition [45].

**Table 1:** Different effectors produced by pathogens and their R genes.

Pathogen	Avr Proteins	R Genes	Host Plant	References
**Bacterial effectors and R genes**				
*Pseudomonas syringae* and *Erwinia amylovora*	AvrB AvrC AvrRpm1 AvrPpiA1 AvrPpiB1 AvrPphD AvrRps4 (AvrPpiE) AvrPto	*Rpg1-b, Rpg2* *Rpm1* *Rpg3* *Rpm1* *Rpm1* *R2* *R3* *R5*	Soybean *Arabidopsis thaliana (A. thaliana)* Soybean *A. thaliana* *A. thaliana* Pea Pea Pea	[46,47,48] [46,49,50,51,52,53] [54,55] [56] [46,51,57,58,59] [54,60,61,62] [46,53,63,64,65,66] [62,67,68,69,70]
	AvrPtoB AvrRpt2 AvrRps4 AvrD HopPtoD2 AvrE AvrF HopAR1 (AvrPphB) HopX (AvrPphE) AvrPphF AvrPphA virPphA	*Rps4* *Pto (and PRF)* *Pto (and PRF)* *Rps2* *Rps2* *Rps4* *Rpg4* *-* *DspA* (*dspEF*) *Rps5 (and Pbs1)* *R3* *R2* *R1*	*A. thaliana* Tomato Tomato Soybean *A. thaliana* *A. thaliana* Soybean - - *A. thaliana* Bean Bean Bean	
*Xanthomonas axonopodis*	AvrBs1	*Bs1*	Pepper	[62,71,72]
		*Xv3* *Bs3* *Bs4* *Rxv*	Tomato Pepper Tomato Bean	
*Xanthomonas oryzae*	AvrXa3 AvrXa5 AvrXa7 AvrXa10 AvrXa21 AvrXa27	*Xa3* *Xa5* *Xa7* *Xa10* *Xa21* *Xa27*	Rice Rice Rice Rice Rice Rice	[73,74,75] [62,76,77] - - - -
*Xanthomonas campestris*	Hax3 Hax4	*Bs4* *Bs4*	Tomato	- -

*Xanthomonas campestris*	AvrB6 pthN pthN2	*B1*	Cotton	[62,75,78]
		*-*		[79]
		
*Xanthomonas oryzae*	AvrRxo1	*Rxo1*	Corn	-
*Xanthomonas citri*	pthA	*-*	-	[62,80]
*Xanthomonas campestris*	XopD AvrBsT AvrXv4 AvrBs2	*-* *BsT* *Xv4* *Bs2*	- *A. thaliana* Tomato Pepper	[46,53,81] [46,53] [46,53,82] [62,83]
*Ralstonia solanacearum*	PopP2	*Rrs1-R*	*A. thaliana*	[84]
**Fungal and oomycetes effectors and R genes**				
*Cladosporium fulvum*	Avr2	*Cf-2*	Tomato	[46,85,86]
	Avr4			[46,87,88]
	Avr4E Avr9 Ecp1 Ecp2 Ecp4 Ecp5	*Hcr9-4E* *Cf-9* *Cf-ECP1* *-* *-* *-*		[89] [90,91,92] [91] [91,93] [94] [95]
	Ecp6 Ecp7			
*Leptosphaeria maculans*	AvrLm1	*Rlm1*	Oilseed rape	[96,97]
	AvrLm6 AvrLm4-7	*Rlm6* *Rlm4 and Rlm7*		[97,98] [97]
*Fusarium oxysporum*	Avr1 (Six4)	*I* (*I-1*)	Tomato	[99]
	Avr2 (Six3)	*I-2*		[100]
	Six2 Avr3 (Six1)	*-* *I-3*		
*Magnaporthe oryzae*	Avr-Pita Avr-Pita2 Avr-Pita3	*Pi-ta* *Pi-ta* *-*	Rice Rice Rice	[46,101,102] [103,104,105] [106,107,108,109]
	Pwl1, Pwl2, Pwl3, Pwl4 Ace1 Avr1-CO39 AvrPiz-t AvrPia AvrPii Avr-Pik/km/kp	Avirulence towards weeping lovegrass *Pi33* *Pi-CO39(t)* *Piz-t* *Pia* *Pii* *Pik, Pik-m* and *Pik-p*	Rice Rice Rice Rice Rice Rice Rice	
*Magnaporthe grisea*	AVR2-YAMO, PWL2, PWL1	*-*	Rice (Yashiro-mochi cultivar)Weeping lovegrass	[104,105,110]
*Rhynchosporium secalis*	Nip1	*Rrs-1*	Barley	[111,112]
	Nip2 Nip3	*-* *-*		
*Blumeria graminis*	Avra10 Avrk1	*Mla10* *Mlk1*	Barley Barley	[113]

*Melampsora lini*	AvrL567	*L5, L6* and *L7*	Flax	[114,115]
	AvrM AvrP123 AvrP4	*M* *P, P1, P2* and *P3* *P4*	Flax Flax Flax	
*Hyaloperonospora parasitica*	Atr1Nd^WsB^	*Rpp1Nd* and *Rpp1-WsB*	*A. thaliana*	[116]
	Atr13	*Rpp13*		
*Phytophthora sojae*	Avr1b-1 Avr1a Avr3a Avr3c Pep-13	*Rps1b* *Rps1a* *Rps3a* *Rps3c* *-*	Soybean	[46,117,118]
			-	
*Phytophthora infestans*	Avr3a Avr4 EPI10 EPI11 inf1	*R3a* *R4* *-* *-* *-*	Potato Tomato *Nicotiana benthamiana* - -	[119,120,121,122]

*Phytophthora parasitica*	para1	*-*	-	[123,124]
**Viral effector genes and R genes**				
Turnip crinckle virus Cucumber mosaic virus (CMV) Potato virus X (PVX) Paprika & pepper mild mottle virus Pepper mild mottle Virus Tobacco mosaic virus	*Coat protein*	*Hrt*		[125]
		*Rcy1* *Nx, Rx1, Rx2* *L2,* *L3, L4* *N’*	*A. thaliana* *A. thaliana* Potato Pepper Tobacco	
Beet necrotic yellow vein virus	*P25 protein*	*Rz-1*	Beet	[125]
CMV TMV	*RNA-dependent RNA* *polymerase*	*RT4-4, Cry* *Tm-1*	French bean Tomato	[125]
PVX TMV	*Movement protein*	*Nb* *Tm-2, Tm-2^2^*	Potato Tomato	[125]

The initial step in signal transduction in the ETI is the recognition and identification of Avr and R proteins which often crosstalk with the PTI. For instance, the R protein of *Arabidopsis*, RRS1-R interacts with the effector protein, PopP2 (TIR NB-LRR) type effector with an extension of WRKY at the C-terminal when released by the bacterium, *Ralstonia solanacearum*. This RRS1-R- PopP2 complex is then translocated into the nucleus for the regulation of other downstream pathways [44]. An avirulent bacterium, *P. syringae* (*Pst*) DC3000 (avrRpt2), activates the resistance to *P. syringae* 2 (RPS2)-dependent ETI in wild-type plants, whereas it is not effective to two *Arabidopsis* PRR and co-receptor mutants, namely *fls2 efr cerk1* (*fec*) and *bak1 bkk1 cerk1* (*bbc*) [45], showing that the PRR and co-receptors play an important role in ETI signaling.

Plant DAMPs were identified in *Arabidopsis* as HMGB protein AtHMGB3 [8]. In general, all plants have HMGB1-related proteins and *Arabidopsis* possess 15 genes that encode HMG-box domain-containing proteins [126]. They have been subdivided into four groups: (i) HMGB-type proteins, (ii) A/T-rich interaction domain (ARID)-HMG proteins, (iii) 3xHMG proteins that contain three HMG boxes, and (iv) the structure-specific recognition protein 1 (SSRP1) [126]. Based on their nuclear location and domain structure, the eight HMGB-type proteins (HMGB1/2/3/4/5/6/12/14) are thought to function as architectural chromosomal proteins, similar to mammalian HMGB1. Notably, AtHMGB2/3/4, the HMGB type proteins, are present in the cytoplasm and as well as the nucleus [126,127]. The cytoplasmic function of these proteins is not yet known. However, the cytoplasmic subpopulations should have greater access to the extracellular space (apoplast) after cellular damage as compared to the AtHMGBs located exclusively in the nucleus [126,127], since they are not bound to DNA and need only cross the plasma membrane to enter the apoplast. The subpopulation of AtHMGB3 raised the possibility that this protein serves a similar function as that of DAMP since recombinant AtHMGB3 was infiltrated into *Arabidopsis* leaves and exhibited DAMP-like activities similar to those of AtPep1, upon treatment with either protein induced MAPK activation, callose deposition, defense-related gene expression, and enhanced resistance to necrotrophic *Botrytis cinerea* [8].

Large scale changes in gene expression are found in *Arabidopsis thaliana* by MAPK activation [128]. The chromatin remodeling in *Arabidopsis thaliana* upon challenge with a synthetically produced 22 amino-acid long flagellin peptide (flg22) that mimics the response to bacterial pathogens. Flg22 is recognized in *Arabidopsis* by the plasma membrane leucine-rich repeat-receptor kinase (LRR-RK) FLS2 and activates two MAPK signaling pathways that initiate an array of defense responses, including the production of several hormones, reactive oxygen species, and the induction of a large set of defense genes, processes generally referred to as MAMP-triggered immunity (MTI) [128].

The second kind of immunity is referred to as effector-triggered immunity (ETI), which is conceived by nucleotide-binding oligomerization domain (NOD)-like receptors (NLRs) and resistance (R) genes, which detects the effector molecules produced by the microorganisms [128]. The genetic and molecular evidence suggested that functional NLR pairs exist, and processes like NLR self-association and heteromeric NLR assemblies are key in the triggering of downstream signaling pathways [129]. Furthermore, the versatility and impact of cooperating NLR pairs combined with pathogen sensing are linked to the initiation of defense signaling in both plant and animal immunity, and different NLR receptor molecular configurations provide opportunities for fine-tuning resistance pathways and augmenting the host’s pathogen recognition spectrum to keep pace with rapidly evolving microbial populations [129]. The concept of R genes emanated from Flor’s hypothesis of gene for gene in the case of pathogen-host virulence factors [42]. Furthermore, he suggested that specific sensors for microbial molecules are present in their hosts and did not rule out variability in these R genes being present in only a few plant varieties, and also many R genes do not confer broad-spectrum resistance, specifying resistance to only some races of a particular pathogen species. These are active primarily inside the cell, using the polymorphic NB-LRR protein products encoded by R genes. They are named after their characteristic nucleotide-binding (NB) and leucine-rich repeat (LRR) domains [130]. NB-LRR proteins are broadly related to animal CATERPILLER/NOD/NLR proteins and STAND ATPases [6]. Pathogen effectors from diverse kingdoms are recognized by NB-LRR proteins and activate similar defense responses. Interestingly, NB-LRR-mediated disease resistance is effective against pathogens that can grow only on living host tissue (obligate biotrophs), or hemibiotrophic pathogens, but not against necrotrophic pathogens [131].

It has already been discussed earlier that the virulence strategy of plant cells leads to the synthesis of intracellular resistance (R) proteins, which specifically recognize pathogen effectors of avirulence (Avr) factors and activate ETI. Crucial amongst the ETI triggers are the genes that respond to viral infections in plants. One major component is the cell wall in plants, whose modifications occur in plants during viral infection, is poorly understood at present. A comprehensive study describes the expression of the potato expansin A3 (StEXPA3) and potato extensin 4 (StEXT4) genes in Potato Virus Y NTN (PVY^NTN^)-susceptible and -resistant potato plant interactions [132]. Furthermore, this study indicated that intracellular distribution and abundance of StEXPAs and HRGPs can be differentially regulated, which depends on different types of PVY^NTN^–potato plant interactions and further confirmed the involvement of apoplast and symplast activation as a defense response mechanism [132]. In a heterogeneous mixture of cells at different stages of infection in plants, the relationships between virus accumulation at the given sites and the accompanying host responses pertaining to altered host gene expressions are not well understood currently. However, a significant study pertaining to this revealed that there was substantive altered expression profiles across gradients of virus accumulation within the spanning groups of cells at different stages of infection [133]. Otulak-Kozieł provided novel insight into cell wall reorganization during PVY^NTN^ infection as a response to biotic stress factors and indicated in situ distribution of the hemicellulosic cell wall matrix components for hypersensitive and susceptible potato–PVY^NTN^ interactions [132]. They further described that the hypersensitive reaction led to activation of XTH-Xet5 in the areas of xyloglucan endo-transglycosylase (XET) synthesis, followed by its active transport to cytoplasm, cell wall and vacuoles [134]. Additional studies by Chen et al. (2017) [135] suggested that genes participating in stress responses, transcription, transport and cell wall were found to have changed expression during the PVY infection stage. Their contention is that the signaling and transcription related genes were almost up-regulated at 12 h, 1 or 2 days, while stress response genes were almost up-regulated at a later stage [135]. In essence, the plant immune system is recognized as a complex network wherein the cell wall and its essential protein components play a significant role in cell wall remodeling. Important progress made in research on plant virus impact on cell wall remodeling of insusceptible and resistant plants, demonstrate that the components of cell wall metabolism can affect the spread of the virus as well as activate the apoplast- and symplast-based defense mechanisms [136]. The cell wall-based multi-complex network can be extensively elucidated employing some sophisticated advanced tools, such as atomic force microscopy, computer-based simulations of mechanical properties of their components, electronic tomography for their mutants and many others [136].

## 4. PTI—ETI Mutualism

PTI recognizes conserved patterns, whereas ETI involves the detection of polymorphic effector molecules released from the pathogens into the plant cells [137]. Generally, low-level basal immunity is conferred by the PTI, which is effective against non-adapted pathogens, whereas ETI is more robust immunity to host-adapted pathogens. The pathogens generally employ varied strategies to invade their hosts, therefore, PTI is a common form of immunity in all the plants, which is then followed by the ETI upon recognition of effectors. Recently, it was reported that for effective responses of ETI, PTI should be present. For instance, *P. syringae* strain lacking phytotoxic coronatine and all effectors except AvrRpt2 induced resistance in WT *Arabidopsis*, but not in lines that lacks multiple PRRs/co-receptors. Similarly, it was reported that AvrRps4-induced resistance is lost in a PTI-deficient genotype [138]. These interesting results suggested that PTI must be functional for effective ETI responses. In the case of PTI, for the production of ROS, phosphorylation of cytoplasmic receptor kinase BIK1 and NADPH oxidase (RBOHD) phosphorylation through BIK1 [45,138,139]. The main features include that ETI increases the levels of protein of PTI signaling components, molecular mechanisms remain unelucidated, ETI requires PTI to provide complete resistance and PTI enhances the output of ETI by restricting pathogen proliferation through hypersensitive responses.

## 5. Signaling of Phytohormones

### 5.1. Brassinosteroids

Brassinosteroid (BR) is an endogenous plant hormone found almost in all organs of plants including seeds, fruits, young vegetative tissues and pollen grains and play roles in the proliferation and expansion of cells. It is reported that BRs function in providing resistance against both biotic and abiotic stresses. Disease resistance was conferred to rice and tobacco after the application with BR [140,141]. Similarly, when BR is applied to barley exogenously, it provided resistance against diseases caused by many species of *Fusarium*. Brassinosteroid-insensitive 1 (BRI1) is a cell surface localized LRR serine/threonine (S/T) kinase that perceives the signals in plants. The binding results in the dissociation of BRI1 from the negative regulator BIK1, and activates the co-receptor BRI1-associated receptor kinase 1 (BAK1) and it heterodimerizes with BRI1, leading to the phosphorylation of the BRI1-interacting signaling kinase (BSK1), and thereby the activation of the BSU1 (protein phosphatase) [141,142]. The signal is then transmitted in the cytoplasm and inhibits brassinosteroid-insensitive 2 (BIN2), a protein kinase, which acts as a negative regulator of the BR biosynthetic pathway and activates the transcription of factors like BZR1 and BES1/BZR2. These transcription factors activate the BR-responsive genes by their promoter in the nucleus. Further, the BAK1 is involved in the regulation of microbe-induced cell death and also interacts with various PRRs and is a part of PAMP-triggered immunity (PTI) [141]. Thus, how BR plays many roles during PAMPs from its perception, activation of stress-related genes and production of secondary metabolites.

### 5.2. Ethylene

Ethylene is involved in various roles in plants like in the growth, development, and providing tolerance to many biotic stresses. It is also involved in the regulation of fertilization, senescence, fruit ripening, and organ abscission. Its perception and signal transduction are conserved among the plants, which show its relevance in their development and survival. The autocatalytic mechanism of ethylene synthesis has induced the ethylene itself during stress. The production of ethylene is regulated by MAPK phosphorylation events.

### 5.3. Abscisic Acid

ABA plays a crucial role in germination, dormancy and seed development and is also involved in biotic and abiotic stresses. It works antagonistically with salicylic acid (SA), ethylene (ET) and jasmonates (JA). It promotes the closure of stomata during stress and enhances the ability in providing resistance through callose deposition. On the other hand, it increases the chances of infection through the exogenous application. For instance, it increased the virulence of *P. syringae* pv. *tomato* on *Arabidopsis* plants. It suppressed the accumulation of defense-related genes like *PDF1.2* (*plant defensin 1.2*), *CHI* (*basic chitinase*), *HEL* (*hevein-like protein*), and *LEC* (*lectin-like protein*), thereby increased susceptibility in *Arabidopsis* against the *Fusarium oxysporum*, which causes wilt, and against bacterium, *Erwinia chrysanthemi*, which causes agents of bacterial wilt infections [141]. Induction of HR-like defense responses occurred at the site of *Peronospora parasitica* inoculation in the ABA-deficient mutant (*aba1-1*) of *Arabidopsis*, while another mutant of *Arabidopsis* (*aba3-1*) failed to close stomata upon the perception of elicitor molecules. Thus, ABA is involved in closing of stomata during stress [141].

## 6. Protein Kinase Signaling Impacts Chromatin Reprogramming in Plant Defense Mechanism

Histone acetylation and deacetylation control MAMP-triggered gene expression, and the histone deacetylase HD2B is known to implicate in the reprogramming of defense gene expression and innate immunity [143]. The MAP kinase MPK3 is reported to directly interact with and phosphorylate HD2B, thereby regulating the intra-nuclear compartmentalization and function of the histone deacetylase [128]. To date, a good number of histone modifiers are known (Table 2) that are involved in plant innate immunity. Furthermore, an example of salicylic acid (SA) signaling can be added to the list that plays an essential role in plant pathogen resistance and is controlled partially by the HDAC SIRTUIN2 (SRT2), which represses the expression of several SA biosynthetic genes such as *PAD4* and *SID2* [144]. Here, srt2 mutant *Arabidopsis* plants were reported to be more resistant to pathogen infection than WT control plants, whereas an SRT2 over-expressing line was more susceptible. In addition, it was reported in *Arabidopsis* that mutations in the HDAC HDA19 result in enhanced basal expression of several biotic responsive genes [145] and improved tolerance to *P. syringae* [143]. Moreover, the rice HDAC HDT701 negatively regulates innate immunity by directly binding and modulating the histone H4 acetylation levels of PRR and defense-related genes [146]. The molecular mechanisms of histone modifications (i.e., methylation, acetylation, and ubiquitination) and chromatin remodeling that contribute to plant immunity against pathogens are interesting areas from a research standpoint and hence needs more elaborate studies pertaining to the subject.

**Table 2:** Shows various histone PTM modifying enzymes conferring sensitivity or resistance to the hosts of pathogens as a result of their actions.

Enzymes	Function	References
Histone deacetylase (HDAC)	Increases sensitivity to *Alternaria brassicicola* and brings about changes in expression of jasmonic acid (JA) and ethylene-regulated genes	[145,147]
	Negatively regulates plant basal defense against the pathogen *Pseudomonas syringae* DC3000	[148,149]
	Negatively regulates the plant basal defense in rice	[150]
Histone methyltransferase	Controls SA/JA pathway genes	[150]
	Faster hypersensitive responses (HRs) to both mutant (hrpA) and pathogenic (DC3000) strains of *P. syringaeand* increased resistance against DC3000	[150,151]
Histone demethylase	Controls systemic acquired resistance (SAR) induction	[152,153]
	Enhances rice resistance to the bacterial blight disease pathogen *Xanthomonas oryzae*	[154]
Histone ubiquitination	Increases sensitivity to *B*. *cinerea* and *A*. *brassicicola*	[155]
Chromatin remodelling factors	Increases resistance to Pst DC3000 in mos1/snc1 background, regulates the expression of R gene SNC1	[156]
	Enhances resistance to Pst DC3000, upregulates the expression of SA-marker genes	[157]
	Increases sensitivity to *B*. *cinerea*, down-regulates expression of ET/JA pathway genes (PDF1.2, VSP2, and Myc2)	[158]

## 7. Chromatin Structure and Modifications

Nucleosome is the packaging structure of the chromosome, which is endowed with a repeated unit of chromatin containing 147 base pairs (bp) of DNA wrapped around a histone octamer, which in turn consists of two copies of the following core histones: H2A, H2B, H3, and H4 and the higher-order chromatin structure formation. Remodeling is achieved by the linker histone, H1, which associates with DNA between two nucleosomes [159]. The globular nucleosome core having the histone tails may undergo diverse post-translational modifications (PTMs), i.e., acetylation, methylation, phosphorylation, ubiquitination, sumoylation, carbonylation, and glycosylation, and through these modifications, can directly affect chromatin structure or can recruit specific “readers or effectors”, to elicit gene regulation during their expression. This is achieved primarily by altering nucleosome stability and positioning, which affect the accessibility for regulatory proteins or protein complexes involved in transcription, DNA replication, and repair [160,161]. Generally, histone acetyltransferases (HATs) mediate transcriptional activation by histone acetylation, while histone deacetylases (HDACs) reverses this process by effecting histone deacetylation [162,163]. Depending on the context of targets, histone methylation and/or ubiquitination can either be an active or repressive marker for transcription in plant-based immunity triggers [164]. Generally, tri-methylations of H3K4 and H3K36 (H3K4me3 and H3K36me3) and mono-ubiquitination of H2B (H2Bub) are supposed to induce gene expressions [164,165], histone methylation of H3K27me3 triggers gene repressions, while H3K9me2 and H4K20me1 are abundant at constitutive heterochromatin and silenced transposons [164,166].

In addition to histone modification, ATP-dependent chromatin-remodeling enzymes are known to use the energy of ATP hydrolysis to remodel chromatin structure by modifying the DNA and histone interactions to dissociate nucleosomes, move histone octamers, and catalyze the incorporation of specific histone variants. ATP-dependent chromatin-remodeling enzymes thus play crucial roles in nucleosome assembly/disassembly and allow the transcriptional machinery to access the DNA [167].

Histone modifications and ATP-dependent chromatin remodeling have only recently attracted attention as potential transcriptional regulators in plant innate immunity. Table 2 summarizes some of such genes and their actions by activation of histone-modifying enzymes leading to an increased or decreased sensitivity in host plants towards pathogens.

## 8. Conclusions and Recommendations

Phytopathogens during their constant interaction with host plants pose a serious threat to their very existence. In order to counter these attacks, the plants develop immunity against them through well-worn strategies, *viz.*, recognizing the pathogens through specific signals and countering pathogenesis through PTI and ETI. During this process, activation of specific receptor proteins plays a key role in pathogen perception and surveillance that trigger signal transduction, initiated in the cytoplasm or at the plasma membrane (PM) surfaces. Plant hosts possess microbe-associated molecular patterns (P/MAMPs), which trigger a complex set of mechanisms through the pattern recognition receptors (PRRs) and resistance (R) genes. These interactions lead to the stimulation of cytoplasmic kinases by many phosphorylating proteins that may also be transcription factors. This entire process is under the control of phytohormones, such as salicylic acid, jasmonic acid and ethylene that are important mediators of defense responses. Histone modifications and their impact on PII is a newly emerging area and hence needs a better understanding. PTMs are smart processes that establish communications between pathogens and plants and alter cell signaling at multiple nodes for the quick reprogramming of the plant for defense responses [168]. Detection of these processes will accelerate our understanding of the regulatory mechanism of plant immunity mediated by PTMs, understanding the molecular processes involved in PII at the cellular and nuclear levels and will thus allow us to design and devise proper scientific interventions that could be useful in augmenting PII under experimental conditions. A field-based survey on these designs is highly recommended for high throughput industrial recommendations and replications of the trials for commercial exploitation.

## Data Availability

Not applicable.
